# Classification of Healthy Subjects and Alzheimer's Disease Patients with Dementia from Cortical Sources of Resting State EEG Rhythms: A Study Using Artificial Neural Networks

**DOI:** 10.3389/fnins.2016.00604

**Published:** 2017-01-26

**Authors:** Antonio I. Triggiani, Vitoantonio Bevilacqua, Antonio Brunetti, Roberta Lizio, Giacomo Tattoli, Fabio Cassano, Andrea Soricelli, Raffaele Ferri, Flavio Nobili, Loreto Gesualdo, Maria R. Barulli, Rosanna Tortelli, Valentina Cardinali, Antonio Giannini, Pantaleo Spagnolo, Silvia Armenise, Fabrizio Stocchi, Grazia Buenza, Gaetano Scianatico, Giancarlo Logroscino, Giordano Lacidogna, Francesco Orzi, Carla Buttinelli, Franco Giubilei, Claudio Del Percio, Giovanni B. Frisoni, Claudio Babiloni

**Affiliations:** ^1^Department of Clinical and Experimental Medicine, University of FoggiaFoggia, Italy; ^2^Department of Electrical and Information Engineering, Polytechnic of BariBari, Italy; ^3^Department of Physiology and Pharmacology “Vittorio Erspamer”, University of Rome “La Sapienza”Rome, Italy; ^4^Department of Neuroscience, IRCCS San Raffaele PisanaRome, Italy; ^5^Department of Integrated Imaging, IRCCS Istituto di Ricerca Diagnostica e NucleareNapoli, Italy; ^6^Department of Motor Sciences and Healthiness, University of Naples ParthenopeNaples, Italy; ^7^Department of Neurology, IRCCS Oasi Institute for Research on Mental Retardation and Brain AgingEnna, Italy; ^8^Clinical Neurology Unit, Department of Neuroscience, University of Genoa and IRCCS Azienda Ospedaliera Universitaria San Martino-ISTGenoa, Italy; ^9^Dipartimento Emergenza e Trapianti d'Organi, University of BariBari, Italy; ^10^Unit of Neurodegenerative Diseases, Department of Clinical Research in Neurology, University of Bari “Aldo Moro”, Pia Fondazione Cardinale G. PanicoLecce, Italy; ^11^Department of Clinical Research in Neurology, University of Bari “Aldo Moro”, Pia Fondazione Cardinale G. PanicoLecce, Italy; ^12^Department of Basic Medical Sciences, Neurosciences and Sense Organs, University of Bari “Aldo Moro”Bari, Italy; ^13^Department of Imaging–Division of Radiology, Hospital “Di Venere”Bari, Italy; ^14^Division of Neuroradiology, “F. Ferrari” HospitalLecce, Italy; ^15^Center for Neuropsychological Research, Institute of Neurology of the Policlinico Gemelli/Catholic University of RomeItaly; ^16^Department of Neuroscience, Mental Health and Sensory Organs, University of Rome “La Sapienza”Rome, Italy; ^17^Laboratory of Epidemiology, Neuroimaging and Telemedicine, IRCCS Centro “S. Giovanni di Dio-F.B.F.”Brescia, Italy; ^18^Memory Clinic and LANVIE–Laboratory of Neuroimaging of Aging, University Hospitals and University of GenevaGeneva, Switzerland

**Keywords:** Alzheimer's disease (AD), electroencephalography (EEG), exact low-resolution brain electromagnetic tomography (eLORETA), linear lagged connectivity, artificial neural networks (ANNs)

## Abstract

Previous evidence showed a 75.5% best accuracy in the classification of 120 Alzheimer's disease (AD) patients with dementia and 100 matched normal elderly (Nold) subjects based on cortical source current density and linear lagged connectivity estimated by eLORETA freeware from resting state eyes-closed electroencephalographic (rsEEG) rhythms (Babiloni et al., 2016a). Specifically, that accuracy was reached using the ratio between occipital delta and alpha1 current density for a linear univariate classifier (receiver operating characteristic curves). Here we tested an innovative approach based on an artificial neural network (ANN) classifier from the same database of rsEEG markers. Frequency bands of interest were delta (2–4 Hz), theta (4–8 Hz Hz), alpha1 (8–10.5 Hz), and alpha2 (10.5–13 Hz). ANN classification showed an accuracy of 77% using the most 4 discriminative rsEEG markers of source current density (parietal theta/alpha 1, temporal theta/alpha 1, occipital theta/alpha 1, and occipital delta/alpha 1). It also showed an accuracy of 72% using the most 4 discriminative rsEEG markers of source lagged linear connectivity (inter-hemispherical occipital delta/alpha 2, intra-hemispherical right parietal-limbic alpha 1, intra-hemispherical left occipital-temporal theta/alpha 1, intra-hemispherical right occipital-temporal theta/alpha 1). With these 8 markers combined, an accuracy of at least 76% was reached. Interestingly, this accuracy based on 8 (linear) rsEEG markers as inputs to ANN was similar to that obtained with a single rsEEG marker (Babiloni et al., 2016a), thus unveiling their information redundancy for classification purposes. In future AD studies, inputs to ANNs should include other classes of independent linear (i.e., directed transfer function) and non-linear (i.e., entropy) rsEEG markers to improve the classification.

## Introduction

Alzheimer's disease (AD) is the most prevalent neurodegenerative disorder affecting the aged people. In AD, a progressive neurodegeneration leads to dementia, characterized by severe cognitive deficits, behavioral symptoms, and loss of autonomy in the daily life (Braak and Braak, 1995).

In the past years, the International Working Group (IWG) and the US National Institute on Aging–Alzheimer's Association (NIA-AA) have proposed an algorithm for the diagnosis of AD based on *in vivo* biomarkers and clinical phenotypes of disease (Förstl and Kurz, 1999; Dubois et al., 2007, 2014; Jack et al., 2010; Albert et al., 2011; McKhann et al., 2011; Sperling et al., 2011). According to the last IWG guidelines (Dubois et al., 2014), diagnostic biomarkers are limited to *pathophysiological markers*. On one hand, these markers include cerebrospinal fluid (CSF) markers, as revealed by the measures of Aβ-42, total tau, and phospho-tau; on the other hand, they include abnormal Aβ-42 and tau accumulation in the brain, as revealed by ligand positron emission tomography (PET) (Förstl and Kurz, 1999). The last IWG guidelines encourage the use of *topographic markers* even if they are not diagnostic. These markers are quite useful to map structural and functional impairment of the brain over time, especially in elderly subjects with initial objective evidence of mild cognitive impairment (MCI) including memory and other cognitive domains but with preserved independence in the daily activities. The *topographic markers* include maps of brain hypometabolism, as revealed by FDG-PET, and maps of brain atrophy and abnormalities of structural and functional brain connectivity, as revealed by structural and functional magnetic resonance imaging (MRI). All those methodologies can capture several processes of disease, but their use is limited because of low availability of the instruments, costs or invasiveness, especially for serial recordings over time.

Keeping in mind the intrinsic limitations of the CSF, MRI, and PET, several independent research groups tested indexes of resting state eyes-closed electroencephalographic (rsEEG) rhythms as candidate topographic markers of AD (Babiloni et al., 2016b). EEG rhythms are the most important feature of collective behavior of brain neural populations and are very relevant for human cognition. Furthermore, EEG procedures are largely available in any country, well tolerated by patients, not affected by subjects' anxiety or task difficulty, and can be repeated over time without habituation effects (Babiloni et al., 2016b).

Previous studies in AD patients and elderly subjects with amnesic MCI have shown that rsEEG may be promising markers for a neurophysiological evaluation of disease status as *topographic markers*. When compared to groups of normal elderly (Nold) subjects, AD groups have been characterized by high power of widespread delta and theta rhythms, as well as by low power of posterior alpha and/or beta rhythms (Dierks et al., 1993, 2000; Huang et al., 2000; Ponomareva et al., 2003; Jeong, 2004).

The use of rsEEG variables as neurophysiologic topographic markers of AD implies that these variables can classify Nold and AD individuals at least with a moderate classification accuracy of 75–80%. In the past years, two papers revised the literature on the accuracy of the classification between AD and Nold individuals by rsEEG features systematically. The article of Jonkman (1997) reviewed 16 studies published in 1983–1995. In those studies, the classification accuracy ranged from 54 to 100% (median of 81%) for the discrimination of the patients with AD dementia and Nold subjects. In the same vein, the paper of Jelic and Kowalski (2009) reviewed 46 studies published in 1980–2008. The classification accuracy ranged from 80 to 85% for the discrimination of the patients with AD dementia (or MCI) and control subjects such as Nold or other forms of dementia.

The present research group has been investigating markers of rsEEG rhythms in MCI, AD, and control subjects in the framework of “BRAINON” program (http://www.brainon.eu). In previous studies of this program, cortical sources of rsEEG rhythms in MCI, AD, and control groups of subjects were estimated by the freeware low-resolution brain electromagnetic tomography (LORETA; Pascual-Marqui et al., 1994). The aim was to enhance spatial information content of scalp-recorded EEG data and to unveil topography of EEG abnormalities associated with AD from prodromal to overt clinical stages (Babiloni et al., 2004, 2008, 2009, 2010, 2011a,b, 2013a,b, 2016b). It was reported that temporal, parietal, and occipital cortical sources of delta and alpha rhythms were altered in AD groups compared with control groups as a function cognitive deficits and abnormalities in brain integrity (Babiloni et al., 2004, 2008, 2009, 2010, 2011a,b, 2013a,b, 2016b).

Recently, our research group tested the hypothesis that Nold and AD individuals with dementia can be discriminated with a moderate accuracy using topographic markers of the rsEEG source current density and functional connectivity (Babiloni et al., 2016a). Results showed a classification accuracy of 75.5% in the discrimination of 120 AD patients with dementia and 100 matched Nold subjects based on cortical source current density (Babiloni et al., 2016a). Of note, this accuracy was obtained by an univariate classifier such as receiver operating characteristic (ROC) curve from the ratio between occipital delta (2–4 Hz) and alpha 1 (8–10.5 Hz) current density. Here we tested if a multivariate classification with artificial neural networks (ANNs) improved that classification accuracy of those original rsEEG markers in AD and Nold individuals. The main issue was whether the combined use of cortical source current density and functional connectivity as inputs of a trained ANN would provide more accurate classifications than those obtained with the two classes of spectral EEG markers considered separately.

## Materials and methods

Details on the subjects, rsEEG database, eLORETA source estimation, and classification with ROC curves were reported in the reference paper quoted in the previous section (Babiloni et al., 2016a). In the following sections, we provide a short description of those methodological procedures for readers' convenience.

### Subjects and diagnostic criteria

The clinical and rsEEG data of the present study refer to 120 AD with dementia and 100 Nold individuals, matched for age, years of education, and gender. Committees of local institutional ethics approved the recording and analysis of EEG data for scientific purposes. Each participant or caregiver subscribed informed consent, in line with the Code of Ethics of the World Medical Association (Declaration of Helsinki).

Probable AD was diagnosed according to the criteria of the Diagnostic and Statistical Manual of Mental Disorders, fourth edition (DSM-IV-TR; American Psychiatric Association) and the National Institute of Neurological Disorders and Stroke–Alzheimer Disease and Related Disorders (NINCDS-ADRDA) working group (McKhann et al., 1984). Individuals underwent medical, neuropsychological, neurological, and psychiatric assessments including Instrumental Activities of Daily Living scale (IADL; Lawton and Brody, 1969), Mini-Mental State Examination (Folstein et al., 1975), Clinical Dementia Rating (CDR; Hughes et al., 1982), and Geriatric Depression Scale (GDS; Yesavage et al., 1983). Exclusion criteria included any kind of evidence of other forms or causes of dementia such as frontotemporal dementia (The Lund and Manchester Groups, 1994), vascular dementia diagnosed according to the criteria of the National Institute of Neurological Disorders and Stroke and Association Internationale pour la Recherché et l'Enseignement en Neurosciences (NINDS-AIREN) working group (Román et al., 1993), Parkinson disease (PD), Dementia with Lewy Bodies (DLB; McKeith et al., 2005), metabolic syndrome, nutritional deficits, tumors, etc. When given, benzodiazepines, antidepressant and/or antihypertensive were suspended for about 24 h before EEG recordings. This procedure did not ensure a complete washout of the drug–longer periods would not have been applicable for obvious ethical reasons- but it made it possible to compare the drug condition across the AD patients. Of note, most of the AD patients (114 out of 120 patients, i.e., 95%) followed a long-term treatment with standard daily doses of acetylcholinesterase inhibitors. In detail, they followed a treatment with donepezil (71 patients; 5–10 mg/die), rivastigmine (29 patients; 10 mg/die) or galantamine (14 patients; 16–36 mg/die).

The Nold subjects underwent medical, neurological, and psychiatric assessments including MMSE (Folstein et al., 1975), Clinical Dementia Rating (CDR; Hughes et al., 1982) and geriatric depression scale (GDS; Yesavage et al., 1983), to exclude from the study subjects with a history of neurological, or psychiatric disorders (including abuse of substances). Finally, a further exclusion criterion was a score in the MMSE lower than 27 for the Nold subjects and higher than 24 for the AD subjects, according to Alzheimer‘s Disease Neuroimaging Initiative (ADNI; http://adni.loni.usc.edu).

Table 1 summarizes some demographic and clinical data of the subjects. *T*-test evaluated the differences (*p* < 0.05, one-tailed) between the groups (Nold and AD) for age, education, MMSE score, and individual alpha frequency (IAF; see below for a description of this index). As expected, a statistically significant difference was found for the MMSE score (*p* < 0.0001; higher MMSE score in the Nold than in the AD group) and for the IAF (*p* < 0.0001; higher IAF in the Nold than in the AD group), while no statistically significant difference was found for age, gender, and education (*p* > 0.05).

**Table 1:** **Demographic and clinical data of normal elderly (Nold) subjects and Alzheimer's disease (AD) patients with dementia**.

	**Gender (female/male)**	**Age (years)**	**Education (years)**	**MMSE (score)**
Nold (*n* = 100)	62/38	69 ± 0.9 SE	9.7 ± 0.4 SE	28.8 ± 0.1 SE
AD (*n* = 120)	78/42	69.8 ± 0.7 SE	9.2 ± 0.4 SE	19 ± 0.3 SE

*Legend: MMSE, Mini Mental State Examination*.

### EEG recordings and preliminary data analysis

During rsEEG recordings, all subjects had to stay with eyes closed in a relaxing state, not moving or talking. 5 min of rsEEG data (EB-Neuro Be-light©, Firenze, Italy) were recorded (128 Hz or higher sampling rate, with a bandpass between 0.01 and 100 Hz) from 19 scalp electrodes positioned over the whole scalp according to the 10–20 System (i.e., Fp1, Fp2, F7, F3, Fz, F4, F8, T3, C3, Cz, C4, T4, T5, P3, Pz, P4, T6, O1, and O2). EEG recordings were performed using frontal cephalic or extracephalic (linked earlobe) reference. Linked earlobe reference electrode was preferred because frontal cephalic reference could attenuate the extracerebral ocular activity on prefrontal (i.e., Fp1, Fp2) and frontal (i.e., F7, F3, Fz, F4, F8) electrodes. However, the use of extracephalic reference was not mandatory to respect the methodological facilities and standard internal protocols of the clinical recording units when the data collection occurred outside a formal clinical trial with a harmonized protocol for the electrode montage. A ground electrode was located between the AFz and Fz electrodes, and scalp electrodes impedances were kept below 5 Kohm. Horizontal and vertical electro-oculographic (EOG) potentials (0.3–70 Hz bandpass) were also recorded to monitor blinking and eye movements.

As a methodological remark, all the mentioned clinical units recorded EEG data with the sampling rate and EEG bandpass set to avoid aliasing. A minority of these rsEEG data (less than 20%) was acquired with 128-Hz sampling frequency. Noteworthy, the use of 128 Hz sampling frequency was sub-optimal for a correct reconstruction of rsEEG signal beyond 40 Hz without aliasing (One has to set a factor from 3 or 4 between the low-pass limit of the analog bandpass filter and the rsEEG sampling frequency).

The EEG data were divided into segments of 2 s and analyzed off-line. The epochs affected by any physiological (ocular, muscular) or non-physiological artifacts were preliminarily identified by an automatic computerized procedure. Two independent experimenters manually checked the artifact-free EEG epochs, before successive analysis. Particular attention was dedicated to the identification of extracerebral contamination of ocular activity (i.e., blinking) in frontal (i.e., F7, F3, Fz, F4, and F8) and prefrontal (Fp1 and Fp2) electrodes, comparing EOG and EEG traces, in EEG recordings with frontal cephalic reference. Finally, all recorded artifact-free EEG data were off-line re-referenced to common average to harmonize the EEG data collected with different reference electrodes.

We computed the spectral power density of the EEG rhythms using a 0.5 Hz frequency resolution Fast Fourier Transform (FFT, Welch algorithm, Hanning window, no phase shift). In line with previous relevant EEG studies (Jelic et al., 1996; Besthorn et al., 1997; Chiaramonti et al., 1997; Babiloni et al., 2005, 2006, 2011b, 2013c), we considered the following standard frequency bands of interest: Delta (2–4 Hz), theta (4–8 Hz), alpha 1 (8–10.5 Hz), alpha 2 (10.5–13 Hz), beta 1 (13–20 Hz), beta 2 (20–30 Hz), and gamma (30–40).

### Cortical sources of rsEEG rhythms as computed by eLORETA

We used the free tool “exact LORETA” (eLORETA) for the linear estimation of the cortical sources activity of rsEEG rhythms in the frequency domain (Pascual-Marqui, 2007a). eLORETA represents the improved version of the previous pieces of software called LORETA (Pascual-Marqui et al., 1994) and standardized LORETA (Pascual-Marqui et al., 2002). Both standardized LORETA and eLORETA showed the same low spatial resolution, with zero localization error in the presence of measurement and biological noise (Pascual-Marqui et al., 2002; Pascual-Marqui, 2007a). However, eLORETA exhibited a better source location in some control parameters (Canuet et al., 2011).

eLORETA uses a head volume conductor model composed of the scalp, skull, and brain. In the scalp compartment, exploring electrodes can be virtually positioned to give EEG data as an input to the source estimation (Jurcak et al., 2007). The brain model is based on a realistic cerebral shape taken from a template typically used in the neuroimaging studies, namely that of the Montreal Neurological Institute (MNI152 template; Mazziotta et al., 1995). The electrical brain source space is formed by 6239 voxels with 5 mm resolution, restricted to cortical gray matter (Fuchs et al., 2002). An equivalent current dipole is located in each voxel. eLORETA solves the so-called EEG inverse problem in the mentioned head volume conductor model estimating “neural” current density values at any cortical voxel for each frequency bin. Input for this regularized inverse estimation (Pascual-Marqui et al., 2002) is the EEG spectral power density computed at all virtual scalp electrodes.

**Figure 1:**
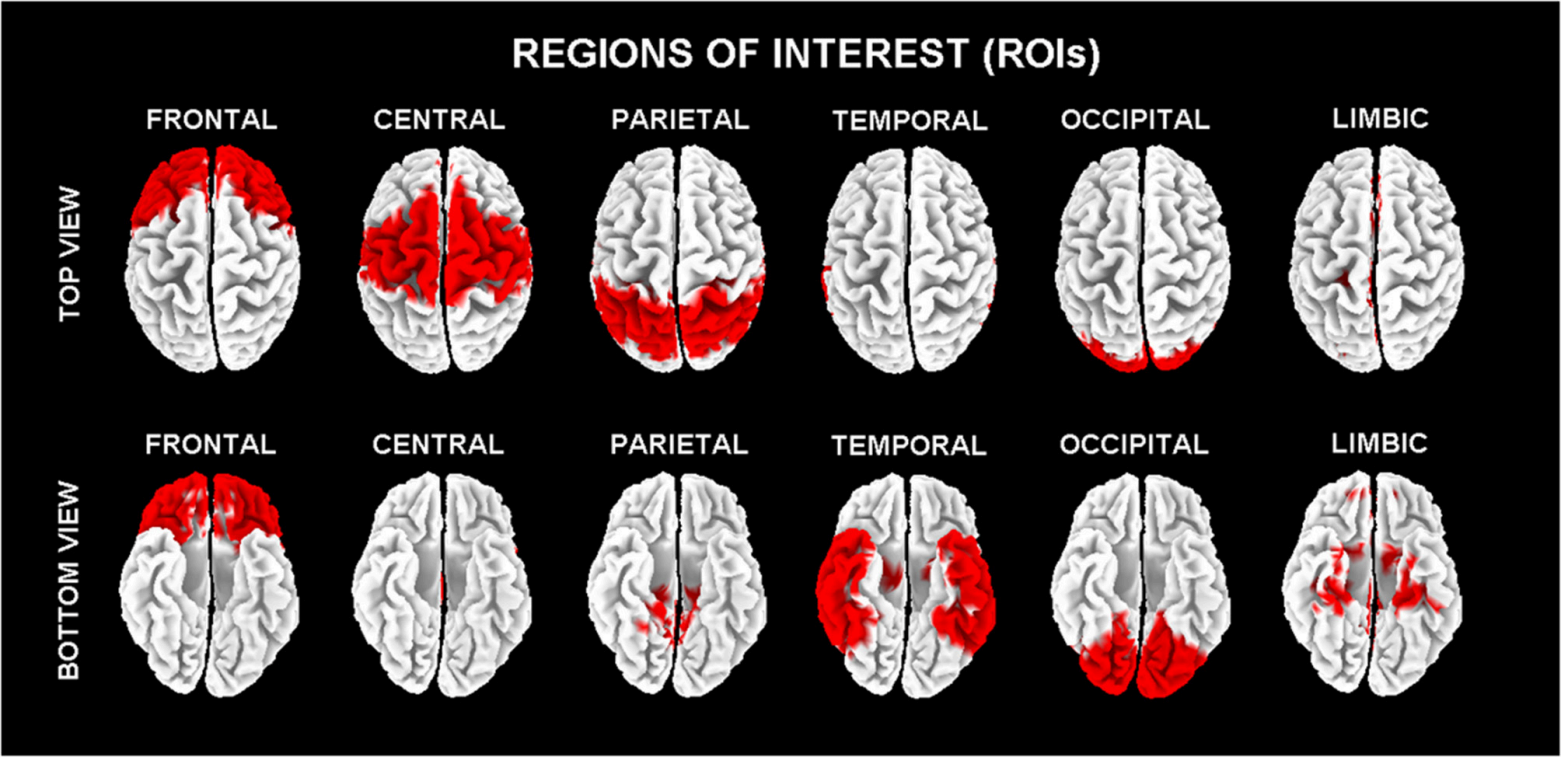
**Regions of interest (ROIs) for the estimation of the cortical sources of resting state eyes-closed electroencephalographic (rsEEG) rhythms by exact low-resolution brain electromagnetic tomography (eLORETA) software**.

In line with the general low spatial resolution of the present EEG methodological approach (i.e., 19 scalp electrodes), the eLORETA solutions were averaged across all voxels in a given cortical macroregion of interest (ROI). The ROIs corresponding to frontal, central, parietal, occipital, temporal, and limbic large regions are shown in Figure 1. These eLORETA solutions were used as rsEEG markers of “cortical source current density reflecting neural synchronization” for the present classification purposes.

eLORETA was also used to estimate the functional connectivity between pairs of ROIs as a measurement of the neural signal communication across distributed populations of cortical neurons in the resting state condition. To this aim, we used the so-called lagged linear connectivity (LLC) tool of eLORETA (Pascual-Marqui, 2007b; Pascual-Marqui et al., 2011). LLC is a linear measure of rsEEG coherence that overcomes the problem of the high phase synchronization and the zero-lag coherence possibly introduced by the procedure of eLORETA source estimation. LLC is also expected to minimize the influence of a third rsEEG source having an influence on the instantaneous coherence between two rsEEG sources not dependent each other (the so-called “common feeding” issue).

For each subject and rsEEG frequency band of interest (i.e., delta, theta, alpha 1, alpha 2, beta 1, beta 2, and gamma), the LLC was computed for 6 ROIs (i.e., frontal, central, parietal, occipital, temporal, and limbic). For the inter-hemispherical analysis, the LLC estimates were calculated between all voxels of the mentioned ROIs of each hemisphere with the corresponding ones of the other hemisphere. The LLC solutions for all voxels of a given pair of ROIs were averaged. For the intra-hemispherical analysis, the LLC estimates were computed for all voxels of a particular ROI with all voxels of another ROI of the same hemisphere. The LLC solutions for all voxels of a given pair of ROIs were averaged, for both the right and the left hemisphere. Those LLC solutions were used as rsEEG markers of “cortical functional connectivity” for the present classification purposes.

### The rsEEG markers used as inputs for artificial neural networks (ANNs)

In the present study, we selected the markers of eLORETA source current density and functional connectivity (LLC) showing a better discrimination between the Nold and AD individuals as revealed by an area under the ROC curves over 70% in the reference study (Babiloni et al., 2016a). According to this criterion, alpha 2, beta 1, beta 2, and gamma rsEEG markers were not considered in the following.

For the eLORETA source current density, we considered 10 markers showing a classification accuracy higher than 75%, namely parietal, temporal, occipital, limbic, central, and frontal theta/alpha1 (i.e., ratio between theta and alpha 1) as well as parietal, temporal, occipital, and limbic delta/alpha1 (i.e., ratio between delta and alpha 1).

For the eLORETA source functional connectivity (LLC), we used 4 markers exhibiting a classification accuracy better than 70%, namely inter-hemispherical occipital delta/alpha 1 as well as intra-hemispherical left occipital-temporal theta/alpha 1, right parietal-limbic alpha 1, and right occipital-temporal theta/alpha 1.

### ANNs: architecture and procedure for the classification of rsEEG markers in Nold and AD individuals

ANN is a mathematical machine learning technique inspired by the core functioning of a biologic nervous system composed of simple processing elements that are interconnected and layered. The main elements of the ANN were inputs, internal, and output layers, which were composed of virtual neurons highly interconnected with each other according to different topologies. Any virtual neuron of a layer was connected to all the neurons of the adjacent layers. Every connection between two virtual neurons was expressed by weight, which represented the “strength” of the connection itself. The weight between each pair of virtual neurons was computed by a learning algorithm along the training phase of ANN, when a set of input examples (training dataset with known classification output) were used as an input to ANN to allow this network to represent the implicit rules that link input features and the classification required as an output (e.g., in the present study, Nold vs. AD individuals). At the end of the training phase, the weights between pairs of neurons represented such rules. In the testing phase, the ability of the ANN to classify was tested in an independent series of datasets.

From a formal point of view, let us indicate with *N*_1_ the number of virtual neurons belonging to the l^th^ layer and with o_k_ the output of the k_th_ neuron of the l^th^ layer. Then, the computation performed by each virtual neuron can be expressed as:

$$\begin{array}{l} net_{k}^{l}\;=\sum_{l\;=\;1}^{{\text{N}}_{\text{l}-1}}{\text{w}}_{\text{kj}}^{\text{l}}{\text{o}}_{\text{j}}^{\text{l}-1}\\ \;o_{k}^{l}\;=f(net_{k}^{l}) \end{array}$$

Where $net_{k}^{l}$ is the weighted sum of the k neurons of the l^th^ layer, w_kj_ is the weight by which the same virtual neuron multiplies the output ${\text{o}}_{\text{j}}^{\left(\text{l}-1\right)}$ of the j^th^ neuron of the previous layer, and f(.) is the so-called activation function, (Basheer and Hajmeer, 2000). The principal activation functions are: log-sigmoid function (logsig), tangent sigmoid function (tansig), saturating linear function (satlin), symmetric saturating linear function (satlins), and pure linear function (purelin).

In the present study, the ANN procedure for the classification of the Nold and AD individuals was formed by the following three steps (see details in Bevilacqua et al., 2016).

In the first step of the procedure, the optimal topology and minimization functions of the ANNs were defined, namely the proper number of ANN hidden layers, the types of activation functions and the number of virtual neurons per any layer, based on the general features of the rsEEG markers in the Nold and AD individuals to be classified. Several approaches could be found in the literature for the search of ANNs best topology using genetic algorithms, e.g., in Bevilacqua et al. (2006). In a previous study (Bevilacqua et al., 2015) a Multi-Objective Genetic Algorithm strategy was proposed to design a robust supervised ANN classifier between AD and NOLD based on EEG markers. Conversely, in this work the search of ANNs best topology was performed by using a Mono-Objective Genetic Algorithm (see details in Bevilacqua et al., 2016) maximizing the mean accuracy calculated on 500 iterations of training, validation and test using random permutations of the input dataset.

Specifically, each “chromosome” modeled all the main features of ANN topology, or rather the number of neurons in the hidden layers (ranging between 1 and 256 for the first hidden layer and from 0 to 255 for the other layers) and their activation functions; in this work, the considered activation functions were the logsig, tansig, purelin, and satlins. Moreover, the activation function set for the neuron in the output layer was the hyperbolic tangent sigmoid (tansig). Before training phase, the whole dataset was standardized using the z-score technique (Zill and Cullen, 2000) whose aim was to rescale data absolute value in an interval centered in 0 and with variance equal to 1.

In the second step of the procedure, the ANNs were trained to determine the connection weights for all virtual neurons and layers. These weights had to optimize the association between the rsEEG source markers in the Nold and AD individual datasets as an input and the correct classification as “Nold” or “AD” in the output virtual neuron. This second step started with the initialization of the connection weights of the virtual neurons by the Nguyen-Widrow's algorithm (Nguyen and Widrow, 1990), which is an efficient method to speed up such a training phase.

After that initialization, the ANNs were trained by a standard supervised learning algorithm namely “Resilient Backpropagation” (Riedmiller and Braun, 1993), adapting the connection weights to minimize the error in the association between the rsEEG source markers in the virtual input neurons and the correct classification as “Nold” or “AD”. In the training phase, the ANN assumed to the best connection weights for all virtual neurons and layers to optimize that association. To this purpose, the whole database of the mentioned rsEEG source markers was divided into three subsets corresponding to 60% (training subset), 20% (validation subset), and 20% (testing subset) of all Nold and AD individual datasets available, respectively. During the second step of the procedure, the training subset was used to train the ANN while the validation subset served to adapt the connection weights of the virtual neurons to avoid the overfitting problem (Witten and Frank, 2005). When the connection weights computed from the training subset produced higher classification errors in the association of the inputs (rsEEG markers) and classification outputs (“Nold” vs. “AD”) in the validation set, the ANN was assumed to fit too much the training set (“overfitting state”). This case was avoided stopping early the ANN training if the network performance fails to improve or remains the same for a number of epochs fixed to 100.

In the third step of the procedure, the performance of the ANNs to classify the Nold and AD individual datasets of the testing subset only (not used in the training and validation phases). That performance in the binary classification (Nold vs. AD) was measured by the following indexes expressed as percentages (%):

1. Sensitivity, defined as the rate of the AD individual testing datasets classified as AD correctly; this index was termed true positive (TP) or TP rate (TPR);
2. Specificity, defined as the rate of the Nold individual testing datasets classified as Nold correctly; this index was termed as true negative (TN) or TN rate (TNR);
3. Accuracy, defined as the sum of the TP and the TN divided by the total number of the individual datasets of the two classes (AD and Nold).

This step-wise classification procedure with training subset, validation subset, and a testing subset was repeated 500 times (iterations). Any iteration produced a value of sensitivity, specificity, and accuracy. The findings reported in the “Results” section refer to the average of the values of sensitivity, specificity, and accuracy over all 500 iterations.

### ANNs: experimental design and statistical analysis

The experimental design aimed at computing the sensitivity, specificity, and accuracy of the classifications of the Nold and AD individual datasets for the following sessions:

1. The 4 most discriminant markers of eLORETA source current density (SCD), namely parietal, temporal, and occipital theta/alpha1 and the occipital delta/alpha 1;
2. The 4 most discriminant markers of eLORETA source functional connectivity (LLC) as listed in the previous section entitled “The rsEEG markers used as inputs for ANNs”;
3. The combination of the mentioned 8 most discriminant markers of eLORETA source current density (SCD) and the functional connectivity (LLC).

**Figure 2:**
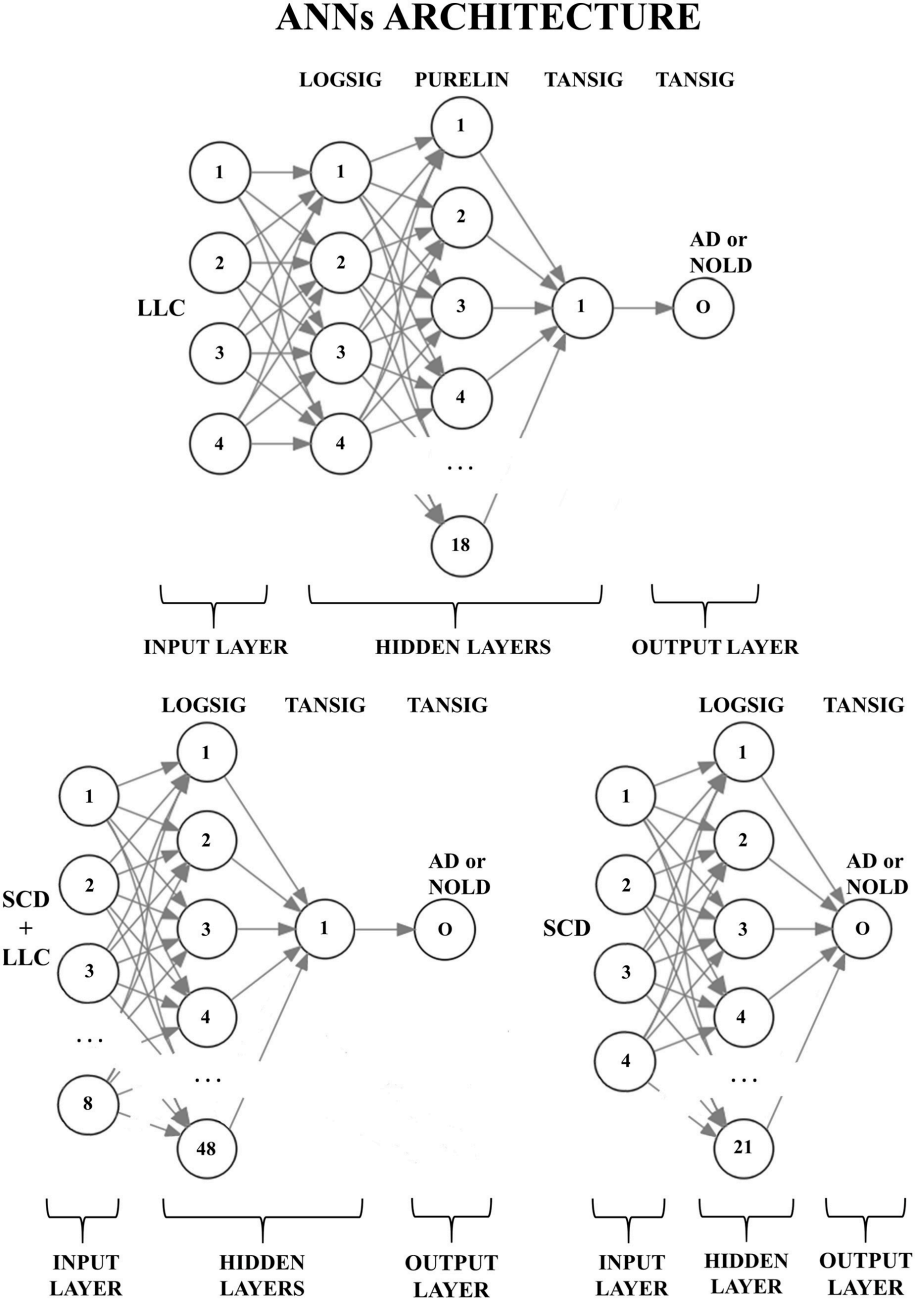
**Structures of the three artificial neural networks (ANNs) used to classify Alzheimer's disease patients with dementia (AD) from Normal elderly subjects (Nold)**. EEG markers are given as inputs in the first layer (input layer); every node (the numbered circles) of every successive layer (i.e., the hidden layers and the output layer) is characterized by an activation function: A non-linear function to decide, in analogy with biological neurons, the output of the node (0 or 1). The output node (O) provides the classification result (AD or Nold). Legend for the input markers: (top) the four best Lagged Linear Connectivity (LLC) markers; (bottom left) the four best LLC markers together with the four best Source Current Density (SCD) markers; (bottom right) the four best SCD markers. Legend for the activation functions: log-sigmoid (logsig), linear (purelin), and tan-sigmoid (tansig).

Figure 2 shows the peculiar architectures of the ANNs used for the above three sessions (they were optimized by the MOGA technique; Bevilacqua et al., 2006). For a given session, the mean and standard deviation of the classification accuracy were computed using the accuracy values of all 500 iterations of that session.

The freeware tool “R” (https://www.r-project.org/) was used to compare the means of the classification accuracy for the following statistical contrasts (*p* < 0.05):

1. The 4 most discriminant markers of eLORETA source current density (SCD) vs. the 4 most discriminant markers of eLORETA source functional connectivity (LLC);
2. The 4 most discriminant markers of eLORETA source current density (SCD) vs. the combination of the mentioned 8 most discriminant markers of eLORETA source current density (SCD) and the functional connectivity (LLC);
3. The 4 most discriminant markers of eLORETA source functional connectivity (LLC) vs. the combination of the mentioned 8 most discriminant markers of eLORETA source current density (SCD) and the functional connectivity (LLC);
4. The 4 most discriminant markers of eLORETA source current density (SCD) vs. the 10 most discriminant markers of eLORETA source current density (SCD).

These statistical contrasts were performed by Shapiro-Wilk normality test (*p* < 0.05). The dependent variable was the classification accuracy while the factor was the Session (4 best discriminant variables of eLORETA source current density, 4 best discriminant variables eLORETA source connectivity, etc.). Dunn's *post-hoc* test was used for multiple comparisons (*p* < 0.05). Tables 2, 3 listed the best 10 markers of SCD and LLC.

**Table 2:** **Results of the classification between single AD and Nold subjects based on composite rsEEG markers of source activity**.

**Source Current Density (SCD)**	**Sensitivity (%)**	**Specificity (%)**	**Accuracy (%)**	**AUROC**
Parietal delta/alpha1	77.5	70	74.1	0.79
Occipital delta/alpha1	73.3	78	75.4	0.82
Temporal delta/alpha1	78.3	70	74.5	0.78
Limbic delta/alpha1	75.8	72	74.1	0.77
Frontal theta/alpha1	70	72	70.9	0.76
Central theta/alpha1	83.3	65	75.0	0.79
Parietal theta/alpha1	78.3	74	76.3	0.82
Occipital theta/alpha1	83.3	68	76.3	0.83
Temporal theta/alpha1	70.8	83	76.3	0.82
Limbic theta/alpha1	83.3	68	76.3	0.81

*Specifically, those composite rsEEG markers were obtained by computing the ratio between the delta and alpha 1 source current density (SCD). The same procedure was followed to form the composite EEG markers obtained by computing the ratio between the theta and alpha 1 SCD. The classification rate is computed by the analysis of the area under the receiver operating characteristic curve (AUROC). The table reports the classification indexes for the composite EEG markers having an AUROC higher than 0.70 (i.e., 70%)*.

**Table 3:** **Results of the classification between single AD and Nold subjects based on composite EEG markers of lagged linear connectivity**.

**Linear Lagged Connectivity (LLC)**	**Sensitivity (%)**	**Specificity (%)**	**Accuracy (%)**	**AUROC**
Intra-hemispheric left parietal limbic	70	74	71.8	0.74
Intra-hemispheric left occipital limbic	73.3	64	69.1	0.71
Intra-hemispheric right temporal limbic	79.2	55	68.2	0.71
Intra-hemispheric right central occipital	80.8	54	68.6	0.70
Intra-hemispheric right parietal occipital	71.7	72	71.8	0.73
Intra-hemispheric right parietal temporal	67.5	71	69.1	0.71
Intra-hemispheric right parietal limbic	68.3	71	69.5	0.72
Intra-hemispheric right occipital temporal	70.8	72	71.3	0.74
Intra-hemispheric right occipital limbic	66.7	76	70.9	0.73
Intra-hemispheric right temporal limbic	76.7	64	70.9	0.73

*Specifically, those composite EEG markers were obtained by computing the ratio between the theta lagged linear connectivity and the alpha 1 linear lagged connectivity, exept the intra-hemispheric right temporal limbic linear lagget connectivity, computed with the ratio between the delta lagged linear connectivity and the alpha 1 linear lagged connectivity. The classification rate is computed by the analysis of area under the receiver operating characteristic curve (AUROC). The table reports the classification indexes for the composite EEG markers having an AUROC higher than 0.70 (i.e., 70%)*.

## Results

### Classification performance of the ANNs

**Figure 3:**
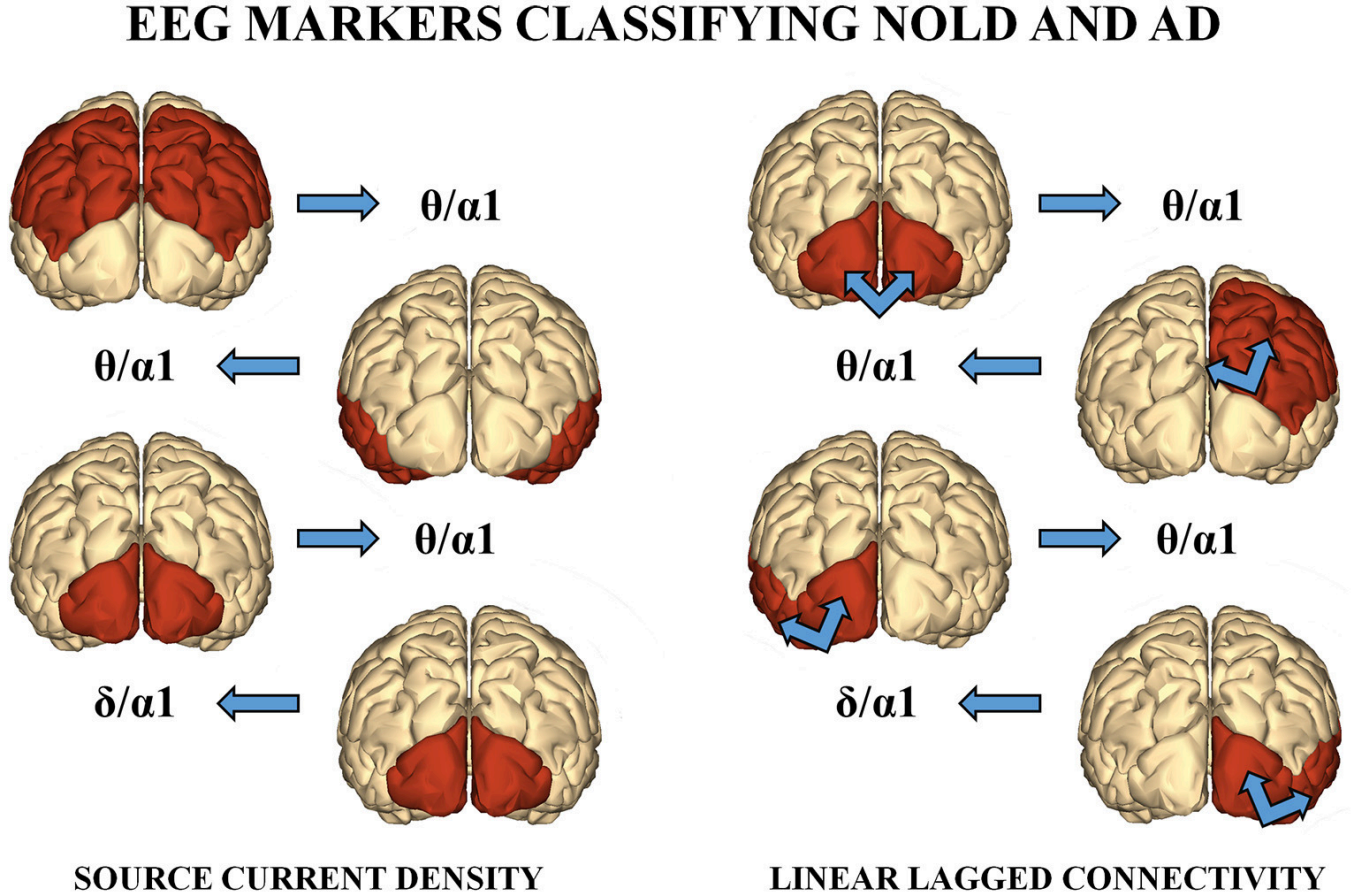
**(Left)** : A topographical representation of the following best 4 discriminant markers of the rsEEG SCD for the classification of Nold and AD individuals. These markers are the following (from the top to the bottom): parietal theta/alpha 1, temporal theta/alpha 1, occipital theta/alpha 1, and occipital delta/alpha 1. **(Right)**: A topographical representation of the following best 4 discriminant markers of the rsEEG LLC for the classification between Nold and AD individuals. These markers are the following (from the top to the bottom): inter-hemispherical occipital delta/alpha 2, intra-hemispherical right parietal-limbic alpha 1, intra-hemispherical left occipital-temporal theta/alpha 1, intra-hemispherical right occipital-temporal theta/alpha 1.

Among the above sessions of ANN classification of the Nold and AD individuals, the best 4 discriminant markers of the rsEEG source current density reached the following best sorting rate: A sensitivity of 79.3%, a specificity of 74.3%, and an accuracy of 77%. Furthermore, the best 4 discriminant markers of the rsEEG source lagged linear connectivity showed a sensitivity of 74.2%, a specificity of 68.9%, and an accuracy of 71.6%. Finally, the combination of the above best 8 discriminant markers of the rsEEG source current density and linear lagged connectivity exhibited a sensitivity of 80%, a specificity of 72.7%, and an accuracy of 76.7%. Table 4 reports these values associated with their standard deviations. Figure 3 illustrates a topographical representation of the best 8 discriminant markers of the rsEEG source current density and linear lagged connectivity. The Shapiro-Wilk normality test (*p* < 0.05) showed that the accuracy values in the 500 iterations of any classification session (i.e., the best 4 discriminant markers of the rsEEG source current density; the best 4 discriminant markers of the rsEEG linear lagged connectivity; the above best 8 discriminant markers) were not Gaussian as distributions. From these distributions, Kruskal-Wallis test disclosed a statistically significant effect (*p* < 0.0001) while Dunn's *post-hoc* test revealed some interesting statistically significant differences in the classification accuracy between session pairs. Specifically, the classification accuracy was higher for the best 4 discriminant markers of rsEEG source current density than the best 4 discriminant markers of rsEEG source linear lagged connectivity (*p* < 0.0001). Furthermore, this accuracy was lower for the best 4 discriminant markers of rsEEG source linear lagged connectivity than the best 8 discriminant markers of the rsEEG source current density and linear lagged connectivity (*p* < 0.0001). In contrast, no difference in the classification accuracy was found between the best 4 discriminant markers of the rsEEG source current density and the best 8 discriminant markers of the rsEEG source current density and linear lagged connectivity (*p* > 0.05).

**Table 4:** **Accuracy, sensibility (true positive rate), and sensitivity (true negative rate) of the ANNs proposed, express as percentage (mean ± standard deviation)**.

	**4 SCD**	**4 LLC**	**4 SCD + 4 LLC**
Sensitivity (%)	79.3 ± 10.6	74.2 ± 11.4	80 ± 10.8
Specificity (%)	74.3 ± 13.2	68.9 ± 14.6	72.7 ± 12.9
Accuracy (%)	77 ± 5	71.6 ± 6.5	76.7 ± 5.2

*Legend: SCD, Source Current Density; LLC, Lagged Linear Connectivity; TPR, True Positive Rate; TNR, True Negative Rate*.

### Control analysis

As reported above, the highest ANN classification of the Nold and AD individuals was obtained with the best 4 discriminant markers of the rsEEG source current density. Therefore, we tested if the 10 best discriminant markers of rsEEG source current density would improve this classification accuracy (Table 2 reports the list of those 10 rsEEG markers). To this aim, the MOGA procedure (Bevilacqua et al., 2006) optimized an ANN with the following features: 1 hidden layer composed of 54 virtual neurons with a LOGSIG activation function; an output neuron with a TANSIG activation function. With this ANN, the best 10 discriminant markers of rsEEG source current density showed a sensitivity of 77.1%, a specificity of 68%, and an accuracy of 74.2%. Again, the distribution of the accuracy values was not Gaussian (Shapiro-Wilk normality test, *p* < 0.05), so we used Wilcoxon test (*p* < 0.05) to evaluate the possible statistically significant differences in the classification of the Nold and AD individuals between the best 10 vs. the best 4 discriminant markers of the rsEEG source current density. Results showed no statistically significant difference (*p* > 0.05).

## Discussion

The present Consortium designed a research program to define and validate rsEEG topographic markers useful to understand the neurophysiological underpinnings of the AD status and the effects of new medications on those underpinnings. Furthermore, the program has to investigate if rsEEG topographic markers can stratify AD individuals based on these neurophysiological bases, namely AD people with “Nold-like” rsEEG markers from those with “AD-like” rsEEG markers. A reliable classification ability of these rsEEG markers would be quite useful in both clinical practice and clinical trials (see Jelic and Kowalski, 2009, for a review). Indeed, AD people with “AD-like” rsEEG markers are expected to have a lower cerebral reserve across the disease evolution. In this line, we have previously used rsEEG markers of the cortical source current density and lagged linear connectivity for the classification of 120 AD patients with dementia and 100 Nold subjects with a linear univariate classifier such as the computation of ROC curves. The results showed that the best classification accuracy of 75.5% was reached using occipital delta/alpha 1 source current density (sensitivity of 73.3% and specificity of 78%). In the present study, we re-analyzed the same database of rsEEG markers with a non-linear multivariate classifier such as the ANN.

The results of the present study showed that the ANN classification produced a classification accuracy of 77% (sensitivity of 79.3% and specificity of 74.3%) using the best 4 discriminant markers of the rsEEG source current density (i.e., occipital, temporal, and parietal theta/alpha 1; occipital delta/alpha 1). Noteworthy, this accuracy was higher than that (71.6%) obtained by the best 4 discriminant markers of the rsEEG source lagged linear connectivity (i.e., inter-hemispherical occipital delta/alpha 1 as well as intra-hemispherical left and right occipital-temporal theta/alpha 1, and right parietal-limbic alpha 1). Furthermore, it did not differ from those reached using the mentioned 8 discriminant rsEEG markers (76.7%) or the best 10 rsEEG markers of source current density (77.1%). Overall, the multivariate non-linear ANN classifiers of the present study reached a moderate classification accuracy that cross-validated that obtained with a standard univariate linear classifier (i.e., ROC curves) from the same database of rsEEG marker (Babiloni et al., 2016a). The same consideration is true for other databases of rsEEG markers of source current density. In previous studies, we have reported that the occipital alpha or the ratio between parietooccipital delta and alpha source current density showed a moderate accuracy of about 75–80% in the classification of Nold subjects and AD patients with dementia (Babiloni et al., 2015, 2016a; Lizio et al., 2015).

The present findings extend to rsEEG markers of source current density and linear lagged connectivity a bulk of previous evidence of other research groups indicating that rsEEG activity provided qualitative markers allowing a moderate discrimination of about 80% between Nold and AD individuals (Brenner et al., 1986, 1988; Hooijer et al., 1990; Strijers et al., 1997; Claus et al., 1999). The present findings also extend the following previous pieces of evidence using quantitative rsEEG markers for classification purposes, mostly from spectral analysis. Huang et al. (2000) combined alpha and theta global field power to reach an accuracy of 84% to classify Nold individuals and AD patients with dementia, and an accuracy of 78% to discriminate the AD patients with dementia and MCI individuals. Adler et al. (2003) reported that the left temporal alpha coherence and the global theta power density returned an accuracy of 80% for the classification of Nold individuals and AD patients with dementia. Moretti et al. (2011) showed that increased global theta/gamma and alpha 3/alpha 2 power density ratios predicted the conversion from MCI to AD or non-AD dementia with an accuracy of 88%. Trambaiolli et al. (2011) described that the temporal modulation of the energy in the delta, theta, alpha, beta, and gamma bands gave an accuracy of 91% in the classification of Nold individuals and AD patients with dementia. Engedal et al. (2015) reported that 20 rsEEG markers (including the alpha frequency peak, total power density, and coherence between electrodes) allowed an accuracy of 90% in the classification of AD patients with dementia from elderly subjects without or with other forms of dementia (e.g., Parkinson's Disease, Dementia with Lewy Bodies). As mentioned in the Introduction section, a full review of the rsEEG classification studies can be found in two articles. One of them (Jonkman, 1997) reported an accuracy ranging from 54 to 100% in the classification of the Nold and AD subjects while the other article (Jelic and Kowalski, 2009) reported an accuracy of about 80–85% between the patients with AD dementia or MCI and the individuals without or with other forms of dementia. Noteworthy, these rates of classification accuracy are in the same order of those obtained from other reliable CSF and neuroimaging biomarkers of AD (Stoeckel et al., 2004; van der Flier, 2005; Klöppel et al., 2008; Álvarez et al., 2009).

Another interesting finding of the present study was that the classification accuracy was not improved combining the (eLORETA) rsEEG markers of the cortical source current density and lagged linear connectivity from the delta, theta, and alpha rhythms recorded during a resting state eyes-closed condition. In this condition of quiet wakefulness, healthy subjects show dominant rsEEG oscillations at about 8–13 Hz in posterior areas of cerebral cortex, the so-called alpha rhythms. These rhythms are associated with a fluctuating cortical inhibition due to a widespread synchronization of activity of cortical pyramidal neurons. These neurons would receive synchronizing signals at around 10 Hz from neurons of thalamocortical, brainstem-cortical, and corticocortical circuits underpinning vigilance and several cognitive functions such as attention and memory (Steriade and Llinás, 1988; Rossini et al., 1991; Neubauer and Freudenthaler, 1995; Klimesch et al., 1996; Klimesch, 1999; Pfurtscheller and Lopes da Silva, 1999; Roh et al., 2011). In the condition of quiet wakefulness, healthy subjects also show low values of delta (<4 Hz) and theta (4–7 Hz) rsEEG rhythms. When delta rhythms reach high power values in that condition, an abnormal tonic “disconnection mode” of the cerebral cortex can be hypothesized. In the quiet wakefulness, these abnormal delta rhythms might have a different neural substrate from sleep delta rhythms (i.e., in stage 4) caused by low-frequency oscillatory signals across cortico-thalamic (<1 Hz) and thalamocortical (<4 Hz) circuits (Steriade, 2003).

Here we report that the classification accuracy based on 8 (linear) rsEEG markers as inputs to ANN (e.g., about 76%) was similar to that obtained with a single rsEEG marker in a previous seminal study on the same database (75.5%; Babiloni et al., 2016a). How can we interpret this finding? From a neurophysiological point of view, the present rsEEG markers of source current density would probe cortical neural synchronization while rsEEG markers of functional connectivity would probe the functional interdependence and efficiency of neurotransmission in different regions of the cerebral cortex. Although these linear rsEEG source markers unveil different relevant neurophysiological mechanisms underpinning low cortical arousal and vigilance in AD patients, they may provide the same core neurophysiological information for the classification of Nold and AD individuals. A high degree of redundancy would prevent an improvement of the classification accuracy either be combining the present linear rsEEG markers of source current density and functional connectivity or increasing the number of rsEEG markers used. To improve the classification accuracy, future studies may use other independent linear and non-linear classes of rsEEG markers as an input to the ANNs, in addition to the present ones. On the one hand, the additional linear markers could be derived from autoregressive models, directed transfer function (DTF), and Granger causality (Granger, 1969; Kaminski and Blinowska, 1991; Kaminski et al., 1997; Korzeniewska et al., 1997; Blinowska, 2011; Katarzyn and Jaroslaw, 2011; Babiloni et al., 2016a). On the other hand, the non-linear ones could be derived from chaos, entropy, and synchronization likelihood (Micheloyannis et al., 1998; Stam, 2005). It can be speculated that those independent mathematical procedures would produce less correlated and redundant input variables for ANNs.

## Conclusions

In a previous study, we showed the best accuracy of 75.5% in the classification of 120 AD patients with dementia and 100 matched Nold subjects based on eLORETA cortical source current density and lagged linear connectivity estimated from rsEEG rhythm (Babiloni et al., 2016a). Specifically, that accuracy was reached using the ratio between occipital delta and alpha1 current density as an input to a linear univariate classifier (i.e., ROC curves). In the present study, we tested the use of ANNs with the same database of eLORETA rsEEG markers. Frequency bands of interest were delta, theta, alpha 1, and alpha 2. Results showed that ANN classification reached an accuracy of 77% using the most 4 discriminative rsEEG markers of source current density (delta/alpha 1 and theta/alpha 1 ratios in posterior cortical lobes). The ANN classification exhibited an accuracy of 72% using the most 4 discriminative rsEEG markers of source lagged linear connectivity (alphas between posterior cortical lobes). With these 8 markers combined, an accuracy of 76% was reached.

Overall, the present results suggest that a non-linear (ANN) multivariate classification rate cross-validated that obtained using a linear univariate classifier in the previous reference study (Babiloni et al., 2016a). Although the linear rsEEG markers of cortical current density and connectivity probe different relevant neurophysiological mechanisms underpinning cortical arousal and vigilance in AD patients, they provide quite redundant information for classification purposes. In future AD studies, inputs to ANNs should combine the present markers with other linear (i.e., directed transfer function, phase lag index) and non-linear (i.e., chaos, entropy, synchronization likelihood) rsEEG markers to improve the classification accuracy of the present moderate values (about 75–80%).

## Author contributions

AT, CBa, RF, RL, CD, and VB gave their contributions to the conception and design of the work. AS, RF, FN, LG, RT, VC, MB, AG, PS, SA, GB, GS, GLo, GLa, FO, CBu, FG, FS and GF were responsible for the enrollments, all the clinical evaluations, and the recordings of EEG data. AT, RL, and CD performed the analysis of data. VB and AB built and trained the Artificial Neural Networks. AT, CD, RL, FC, and GT performed the statistical analysis. AT, CBa, RL, and CD gave their contribution in the interpretation of the results. AT, CD, RL, VB, and CBa contributed in drafting the work and revising it critically for important intellectual content. AS, RF, FN, LG, RT, VC, MB, AG, PS, SA, GB, GS, GLo, GLa, FO, CBu, FG, FS, and GF contributed to the revision of the first and later versions of the manuscript.

### Conflict of interest statement

The authors declare that the research was conducted in the absence of any commercial or financial relationships that could be construed as a potential conflict of interest.
